# Curcumin Protects β-Lactoglobulin Fibril Formation and Fibril-Induced Neurotoxicity in PC12Cells

**DOI:** 10.1371/journal.pone.0133206

**Published:** 2015-07-17

**Authors:** Mansooreh Mazaheri, Ali Akbar Moosavi-Movahedi, Ali Akbar Saboury, Fariba Khodagholi, Fatemeh Shaerzadeh, Nader Sheibani

**Affiliations:** 1 Institute of Biochemistry and Biophysics, University of Tehran, Tehran, Iran; 2 Standard Research Institute, Food Department, Karaj, Iran; 3 Center of Excellence in Biothermodynamics, University of Tehran, Tehran, Iran; 4 Neuro Biology Research Center, Shahid Beheshti University of Medical Sciences, Tehran, Iran; 5 Departments of Ophthalmology and Visual Sciences, University of Wisconsin School of Medicine and Public Health, Madison, Wisconsin, United States of America; Islamic Azad University-Mashhad Branch, Mashhad, ISLAMIC REPUBLIC OF IRAN

## Abstract

In this study the β-lactoglobulin fibrillation, in the presence or absence of lead ions, aflatoxin M1 and curcumin, was evaluated using ThT fluorescence, Circular dichroism spectroscopy and atomic force microscopy. To investigate the toxicity of the different form of β-Lg fibrils, in the presence or absence of above toxins and curcumin, we monitored changes in the level of reactive oxygen species and morphology of the differentiated neuron-like PC12 cells. The cell viability, cell body area, average neurite length, neurite width, number of primary neurites, percent of bipolar cells and node/primary neurite ratios were used to assess the growth and complexity of PC12 cells exposed to different form of β-Lg fibrils. Incubation of β-Lg with curcumin resulted in a significant decrease in ROS levels even in the presence of lead ions and aflatoxin M1. The β-Lg fibrils formed in the presence of lead ions and aflatoxin M1 attenuated the growth and complexity of PC12 cells compared with other form of β-Lg fibrils. However, the adverse effects of these toxins and protein fibrils were negated in the presence of curcumin. Furthermore, the antioxidant and inhibitory effects of curcumin protected PC12 cells against fibril neurotoxicity and enhanced their survival. Thus, curcumin may provide a protective effect toward β-Lg, and perhaps other protein, fibrils mediated neurotoxicity.

## Introduction

Amyloid fibrils are fibrillary protein aggregates that are implicated in a variety of human diseases [1–4]. Some researchers have shown that the amyloid fibril-forming propensity is a generic property of all polypeptides [5]. Certain metal ions contribute to pathogenesis of these degenerative diseases [6]. The generally accepted argument on the role of divalent metals in protein aggregation is based on their ability to act as bridges, as well as to provide an electrostatic screening between the negatively charged groups of the neighboring protein molecules [7]. Indeed, protein aggregation is generally promoted by the electrostatic screening due to the action of monovalent and/or divalent ions [8, 9].

β-lactoglobulin (β-Lg) is a low-molecular-weight whey protein capable of binding and transporting small hydrophobic molecules. β-Lg is an important protein for food industry because of its functional and emulsion stabilizing properties[10]. β-Lg exists in the form of a dimmer at room temperature and neutral pH. However, it dissociates into monomers at acidic pH (<pH 3) due to electrostatic repulsions between the subunits. β-Lgis composed of nine β-strands and one α-helix, in which the hydrophobic sequences are mostly buried [11, 12]. In addition, β-Lg can form fibrils when heated above its denaturation temperature (75°C) under acidic conditions and ionic strength [13]. However, longer treatment at 80°C initiates the oxidation process promoting the exchange between thiol groups and disulphide bridges [14].

Many amyloid fibrils are formed at mild conditions (e.g. 37°C and pH 7), while fibril formation of β-Lg can only occur under extreme conditions (heating at 75°C or above of it, acidic pH or by dissolving in high molarities of urea) [15, 16]. Thus, β-Lg needs to be hydrolyzed in order to be incorporated into fibrils [15]. β-Lg is one of the proteins that used in food industry because of its fibrils properties. The fibrils of β-Lg can increase viscosity and used for microcapsulation [17].

Lead (Pb) ions can lead to toxic effects regardless of the exposure pathway, and interact with proteins with sulfhydryl, amine, phosphate and carboxyl groups [18]. There are many possible ways in which toxic metal exposure could lead to increased risk of Parkinson’s disease. One of the most important effects of lead poisoning is the induction of oxidative stress via the production of free radicals [19]. Lead is one of the important contaminants found in milk. So, Codex as an international organization for developing food standards was set the maximum limit for lead in milk [20]. The interaction between β-Lg and lead ion studied by some researchers and it is reported that β-Lg can interact with lead ions without any considerable change in the protein structure [21].

Aflatoxin M1 (AFM1) is the hydroxylated aflatoxin metabolite present in milk products obtained from livestock fed a diet contaminated with aflatoxin B1. The affinity of AFM1 toward the milk protein fractions was investigated by Govaris et al. in 2001 which regarded the distribution of AFM1 between whey and curd during cheese manufacturing [22]. A partition of AFM1 occurs between whey and curd, with 40–60% of the total AFM1 amount is registered in the whey compared with that present in the milk [22–26]. Also it is reported that the interaction of AFM1 with whey proteins can happen and cannot be ignored, especially considering the possibility to reuse the protein fraction as a raw material in other processes [27]. So we proposed that AFM1 enters into the whey protein and interacts with the β-Lg affecting its fibrillation.

Curcumin, a main coloring substance from the root of the plant *Curcuma longa Linn*, has potent antioxidant properties for ROS protection [28]. It can suppress oxidative damage, inflammation, cognitive deficits, and amyloid accumulation. In some researches, it is reported that curcumin may be able to participate in the treatment of Alzheimer's disease through several different molecular mechanisms. Some results presented provide insight into the different mechanisms of curcumin chelation and peptide binding and show that curcumin can both bind to some proteins and also chelate metal ions [29]. It is reported that some metal ions-curcumin complexes inhibits fibril formation and can remove the pre-formed amyloid deposits [29, 30].

Forster energy transfer measurements and molecular docking studies suggested that curcumin binds to the central calyx of β-Lg [31].The interaction of β-Lg with curcumin does not affect β-Lg conformation or its state of association [31]. The complex formation of curcumin with to β–Lg has been investigated employing spectroscopic techniques and it reported that β–Lg interacts with curcumin with an association constant of 1.04 ± 0.1 × 10^5^ M^-1^ [31]. Curcumin binds through multiple forces, directly to some molecules such as metal ions. Electrochemical studies as well as IR and UV analysis suggested a strong ligand interaction between Pb^2+^ and curcumin [32,33]. The functional groups on curcumin are suitable for interaction with the phenyl rings [33], and AFM1 has a phenyl ring in its structure. Here the β-Lg fibrillation, ROS generation during fibrillation process, and the protective effects of curcumin on these processes were investigated. We also determined the viability and complexity of the PC12 cells incubated with native and fibril forms of β-Lg prepared in the presence of Pb^2+^, AFM1, and curcumin.

## Materials and Methods

### Materials

Bovine β-Lg type A (L7870),Curcumin (C-1386), thioflavin-T (ThT; T-3516), AFM1, Nerve growth factor (NGF) and Dulbecco’s modified Eagle’s medium (DMEM) were purchased from Sigma-Aldrich (USA). Fetal bovine serum (FBS) and the horse serum were obtained from Gibco (Life Technologies, USA) and Rat pheochromocytoma PC12 cells from Pasture Institute of Iran (Tehran, Iran) was used. Pb standard (1.09969, Titrisol 1000 mg, Lead (ІІ) nitrate in water) and luminol (5-amino-2, 3-dihydro-phthalazine-1, 4-dione) were from Merck (Germany). A stock solution of 1000 ppm Pb (NO_3_)_2_ in water, and a stock solution of 100 ng/mL(ppb) of AFM1 in methanol were used. All other materials and reagents were of analytical grade, and double distilled water was used for preparation of solutions.

### Preparation of β-Lg, Curcumin, AFM1 and Pb Solutions

β-Lg (10 mg/mL) was dissolved in HCl solution (pH 2.0). After filtration, the exact concentration of protein was determined by UV spectrum of β-Lg using molecular absorption coefficient of ε_278 nm_ = 17,600 M^−1^ cm^−1^ [34]. Curcumin solution was prepared in methanol, and the exact concentration was measured by determining light absorption at theε_420 nm_ = 48,000M^-1^cm^-1^ [35]. Curcumin solution was freshly diluted in the protein buffer. Before fibrillation process, AFM1 and Pb solutions were dried by nitrogen gas and diluted in buffer before adding to the protein stock solution to desired concentrations. For fibrillation experiments, the protein was diluted to 5 mg/mL. Here the concentration of AFM1 was considered 0.5 ng/mL (ppb) according to the maximum limit of AFM1 in milk in codex [20] and also 1 ppb. The Pb^+2^ concentration was 0.2 ppm, and was chosen based on Joint FAO/WHO Expert Committee on Food Additives (JECFA) evaluation that reported the levels of lead in blood of the majority of occupationally unexposed people are between 150–250 ng/mL for adults and children [36]. The mole ratio for curcumin: β-Lg was 1. The mole ratios for AFM1: β-Lg was 4.85 ×10−^6^ and 9.7 ×10−^6^, and for Pb ion: β-Lg was 3.5×10 ^-3^.The samples were placed in a shaking water bath at 80°C with a shaking speed of 290 rpm for 0 to 24 h.

### Methods

The behavior of β-Lg fibrillation was studied under several conditions. First: the fibril formation of protein (5 mg/mL) in the absence or presence of curcumin at 80°C and pH 2.0. Second: fibrillation of the protein in the presence of AFM1 or Pb^2+^. Third: fibrillation of the protein in the presence of aforementioned toxins with or without curcumin. Also, the effect of concentration of residual methanol after curcumin dilution on β-Lg fibril formation without curcumin was studied and no inhibitory or accelerating effect of methanol was noted.

#### ThT fluorescence assay of β-Lg fibrillization

ThT has the ability to bind to amyloid fibrils. Thus, the ThT fluorescence assay was used to measure the conversion of protein into fibrils after heating the protein solution. For ThT fluorescence assays, some samples were removed after the certain incubation time. A ThT stock solution 3.0 mM in phosphate buffer (10 mM Phosphate, 150 mM NaCl, pH 7.0) was filtered through a 0.2 μm filter and was diluted 50 times in a phosphate buffer before use. After heating the protein solutions, 48μL of the fibril samples were mixed with 4 mL of ThT solution and allowed to bind to the ThT for 1 min. The fluorescence measurements were carried out using a fluorescence spectrophotometer (Cary Eclipse fluorescence spectrophotometer, model 100) at 482 nm and excitation wavelength of 460 nm, and emission spectrum between 470 and 500 nm. The fluorescence intensity at 482 nm was compared for β-Lg solutions incubated with or without curcumin, AFM1 and Pb^+2^. For the fluorescence measurements, the assay for each sample was repeated three times.

#### Circular dichroism(CD) spectroscopy

For analyzing the secondary structure of samples after fibrillation, CD spectra were recorded on a circular dichroism spectrometer (model 215, Aviv, USA) at 25°C using a bandwidth of 1 nm, a step interval of 1 nm, and a slit width of 0.02 mm. A quartz cell with a 1 mm path length was used for far-UV measurements. Three scans of duplicate samples were measured and averaged. Control buffer scans were run in duplicate, averaged, and then subtracted from the sample spectra.

#### Atomic force microscopy (AFM)

AFM is a powerful tool to study the structure of amyloid fibrils. AFM was used to monitor the impact of curcumin on β-Lg fibril formation. For AFM analysis the protein samples were diluted to 0.2 mg/mL in buffer and 10 μL of each sample was placed on a piece of mica (1 cm×1 cm pieces). The AFM samples were dried at room temperature and then used for AFM images. AFM images were recorded by AFM-NSOM (Thermo microscopes Autoprob; Vecco Company).

#### Determination of Reactive Oxygen Species (ROS) production

To assess ROS production, Luminol-derived chemiluminescence was used by Synergy H4. The level of ROS was determined after β-Lg fibrillation in the absence or presence of curcumin, AFM1 or Pb ions. These experiments were performed by adding 235 μL of carbonate buffer (pH 11, 0.2 M) and adequate amount of luminal to each well and repeated three times.

#### Cell culture

For cell culture studies, we used the method that Kachioei et al. used [37]. According to this method, PC12 cells were grown in DMEM supplemented with serum (10% (v/v) horse serum and 5% (v/v) FBS), and 1% antibiotic mixture comprising penicillin-streptomycin in a 5% CO2 incubator with humidified atmosphere at 37° C. The medium was carefully changed three times a week. By incubating with NGF (50 ng/mL), the cells were differentiated every day for 6 days.

#### MTT reduction assay, andanalysis of Neurite Outgrowth of PC12 Cells

Differentiated PC12 cells, seeded in 96 wells plates, were incubated with β-Lg without any treatment and β-Lg fibril samples obtained in the absence and/or presence of curcumin, Pb ions and AFM1. All the protein samples were diluted 200 times with sterile DMEM/F12 medium to have 0.025 mg/mL β-Lg in the medium. The diluted samples were added to the PC12 cells in the 96-well plates, from which the medium was removed. The sample solutions were incubated with PC12 cells for 4 and 8 h. Cells were incubated with 3, (4, 5-dimethylthiazol-2-yl) 2, 5- diphenyl tetrazolium bromide (MTT solution). The Cell viability was measured using MTT reduction assay. MTT is reduced to blue formazan crystals in living cells. Losing the ability to convert MTT into formazan is indicative of cellular redox changes that could result in toxicity [38]. MTT reduction was assessed 4 and 8 h after incubation. MTT was added to the culture medium to yield a final concentration of 4 mg/mL. The cells were incubated with the MTT for 4 h in a CO_2_ incubator; then 100 μL of dimethyl sulphoxide was added to dissolve the formazan crystals. The absorbance at 630 nm was then read using a microplate reader. Cell viability was reported relative to control cells that were not exposed to the fibril solutions. The experiments were repeated and all data are expressed as means ± standard error of mean (S.E.M.) for n independent determinations.

The morphology of the samples was investigated by phase contrast microscopy (Olympus, IX71). As we cited in previous publication [37], two random images were acquired from each well. The criteria were the cell body and a minimum of 50 cells per treatment were quantified. The cell body of individual cell was distinct from neighboring cell bodies and processes were completely within the field of view. The average neurite length, cell body area, neurite width, and the number of primary neurites and bipolar morphology were quantified. The cell area that neurite processes originated from and contained cell organelles was defined as cell body area.

By summing the lengths of the primary process and all branches, the neurite length was calculated. For determination of the average neurite width, a Cell A software was used for cell body area calculation and the results divided by the length of the neurite. Clear protrusions from the cell body with length greater than 10 μm, defined as primary neurites. Bipolar cell was described as a cell body with one process at each end. In order to evaluate neurite networks, images were analyzed using the cell counter plug-in to score all branching nodes in each image. The sites with individual neurites branch or separate neurites contacted each other were defined as nodes.

The results of MTT reduction assay and analysis of Neurite Outgrowth of PC12 Cells were expressed as mean ± S.E.M. (Standard Error of Mean) and processed by commercially available software Graph Pad Prism 5.0. One-way ANOVA followed by post-hoc analysis was performed for cellular tests. P-value less than 0.05 (P<0.05) was considered to be statistically significant (* P<0.05, ** P<0.01, and ***P<0.001).

## Results and Discussion

The first step in protein aggregation, in the majority of cases, is partial unfolding. The hydrogen bonds in the protein are weakened by increasing the temperature; while the hydrophobic interactions are strengthened and activate the aggregation process [39–41]. The protein thiol groups are normally inaccessible in the native form, and partial unfolding of the protein exposes the thiol groups. The reactive thiol groups react via a thiol/disulfide exchange reaction with one of the two intra‐molecular disulfide bonds of a non‐reactive protein molecule, and an intermolecular disulfide bond is formed. It was shown that peptides, and not the intact monomers, are the building blocks of the fibrils derived from β-lg heated at 80°C and pH 2 [15]. Thus, β-lg has to be hydrolyzed first, and then fibril formation can occur.

### Fluorescence studies of the effects of curcumin, Pb ions, and AFM1 on the β-Lg fibril formation

**Fig 1:**
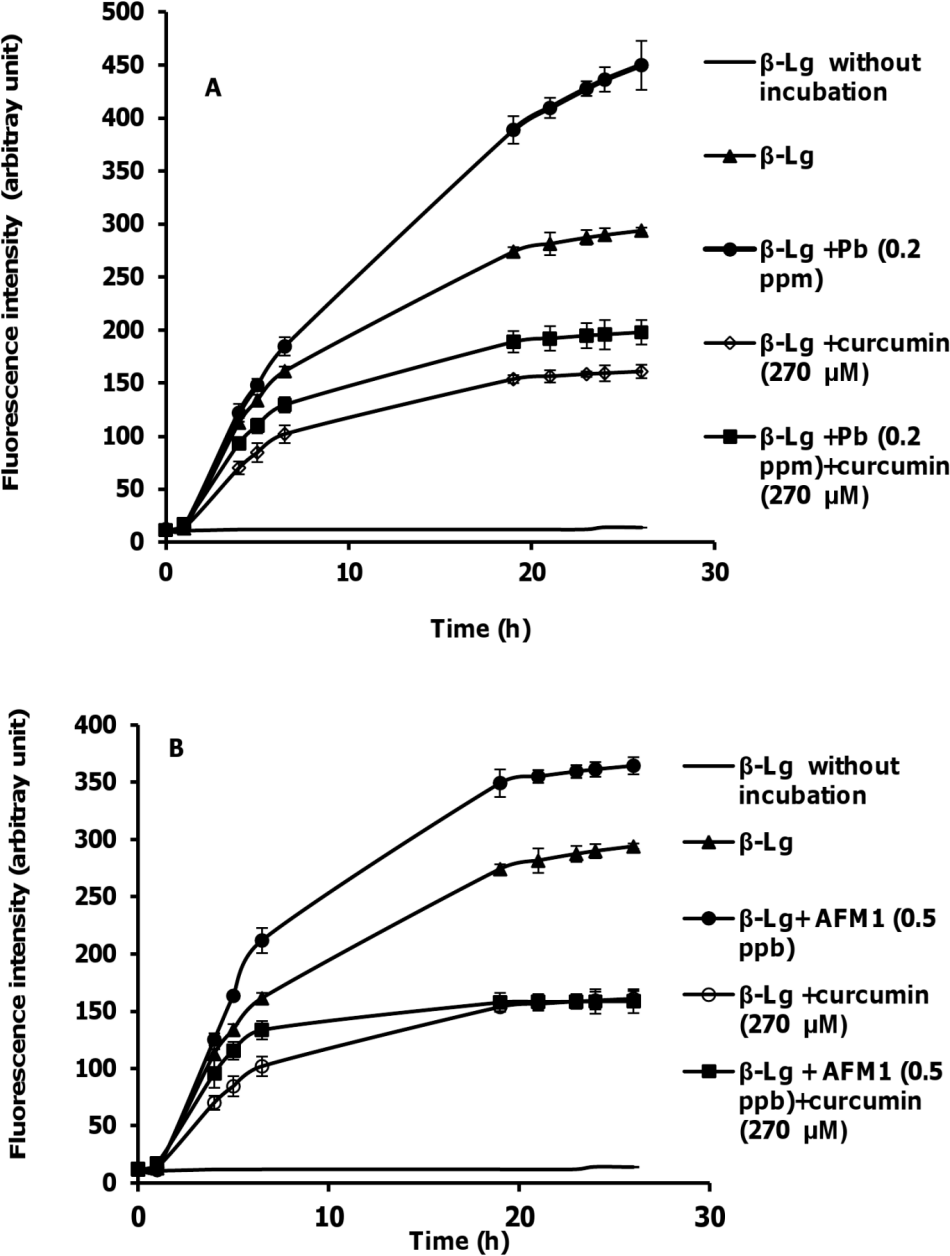
ThT fluorescence of β-Lg (5 mg/mL) at pH 2 heated at 80°C. (A) In the absence and presence of Pb^2+^, curcumin and their mixture. (B) In the absence and presence of AFM1, curcumin and their mixture. Data represent the means ± SD of 3 independent measurements.

ThT binding was used as an indicator of β-Lg fibril formation. As depicted in the inset of Fig 1, there is a short lag phase with duration of approximately 2 h, during which the fluorescence remained low. The ThT fluorescence intensity of β-Lg increased and reached a maximum level at ∼24 h. The effects of curcumin, Pb ions, and AFM1 on the β-Lg amyloid fibrillation were first examined by ThT fluorescence measurements. In Fig 1A and 1B, the ThT fluorescence of β-Lg by itself increased significantly in the following hours but finally reached a constant level. In contrast, our results revealed a decrease in the level of β-Lg fibrillation in the presence of curcumin. However, incubation of β-Lg in the presence of Pb^2+^ accelerated the rate of protein fibril formation. The ThT fluorescence intensity after incubation of β-Lg in the presence of AFM1 exhibited a greater increase compared with β-Lg alone. It was apparent that the presence of curcumin resulted in a slower growth rate compared with control. Our data also indicated that β-Lg samples containing toxins with curcumin had significantly lower final ThT fluorescence intensity than β-Lg sample containing toxins without curcumin. Thus, curcumin protected β-Lg from the toxins (Pb^2+^ and AFM1) induced fibril formation.

### Secondary structure changes

**Fig 2:**
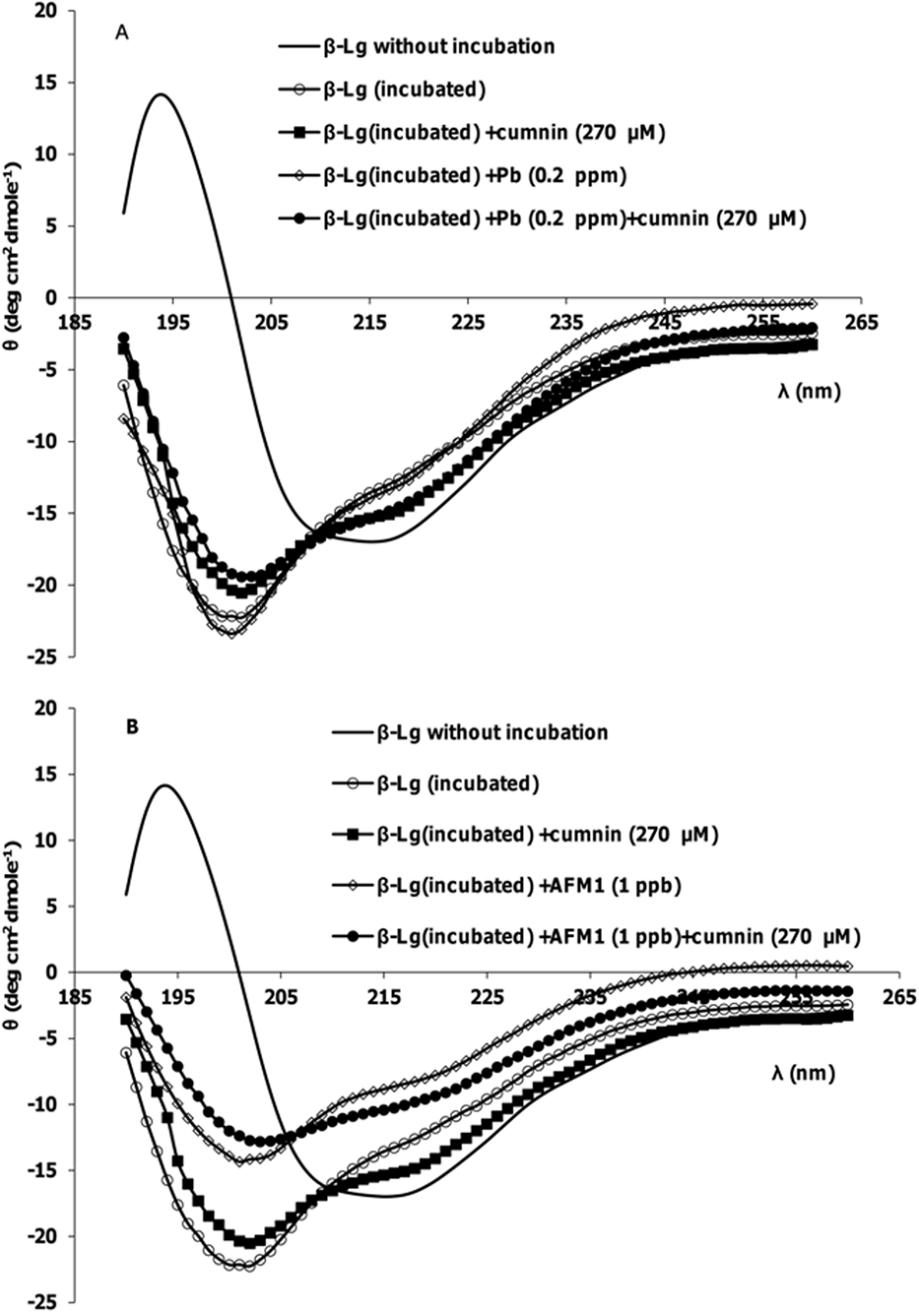
Representative far-UV CD spectra of β-Lg samples after incubation at pH 2 and 80°C. (A) In the absence and presence of Pb^2+^, curcumin and their mixture. (B) In the absence and presence of AFM1, curcumin and their mixture.

Fig 2, A and B show far-UV CD spectra of β-Lg after incubation. Minima of spectra between 208 and 222 nm is characteristic of helical structure. As shown in Fig 2, after incubation at 80⁰C, this minimum disappears. The intensity loss of the 222 nm band indicates that the increase in random coil/β-sheet conformations is accompanied by a reduction in the α-helical content of the protein structure. On the other hand, the significant differences in far-UV CD spectra are observed when β-Lg incubated with AFM1. This change is less in the presence of curcumin. It is shown that the bases of β-Lg fibrils are peptides [15]. In fibrillation process of β-Lg at 80°C and acidic hydrolysis, the cleavage of the bonds before or after aspartic acid residues in β-Lg occurs and small peptides were formed. But only the specific peptides with a high hydrophobicity can form fibrils. So there are the mixture of non-aggregated peptides and fibrils in this solution [15]. The CD spectra of incubated β-Lg samples verified their results. Also there are different reports about quenching ThT by curcumin and its effect on the fluorescence of ThT. In some researches it is shown that curcumin can decrease ThT fluorescence in some concentration [42]. The reason of this effect was referred to the excitation wavelength of ThT and absorption spectrum of curcumin, which are near. So when there are mixture of the ThT and curcumin, it is possible to see inner filter effects. ThT is used regularly to quantify the fibril formation and detect of inhibitory effects of some compounds. But the results can be verified by using other techniques such as AFM images or CD spectra. The CD spectra approved our comment about inhibition effect of curcumin, because in the presence of curcumin, the change of the secondary structure of β-Lg after incubation is less compared with incubation of the protein without curcumin.The fibrils are stabilized by β-sheets held together by hydrogen bonds, and curcumin may inhibit hydrogen bonding between β-sheets.

### The effects of curcumin, Pb ions, and AFM1 on fibril morphology

**Fig 3:**
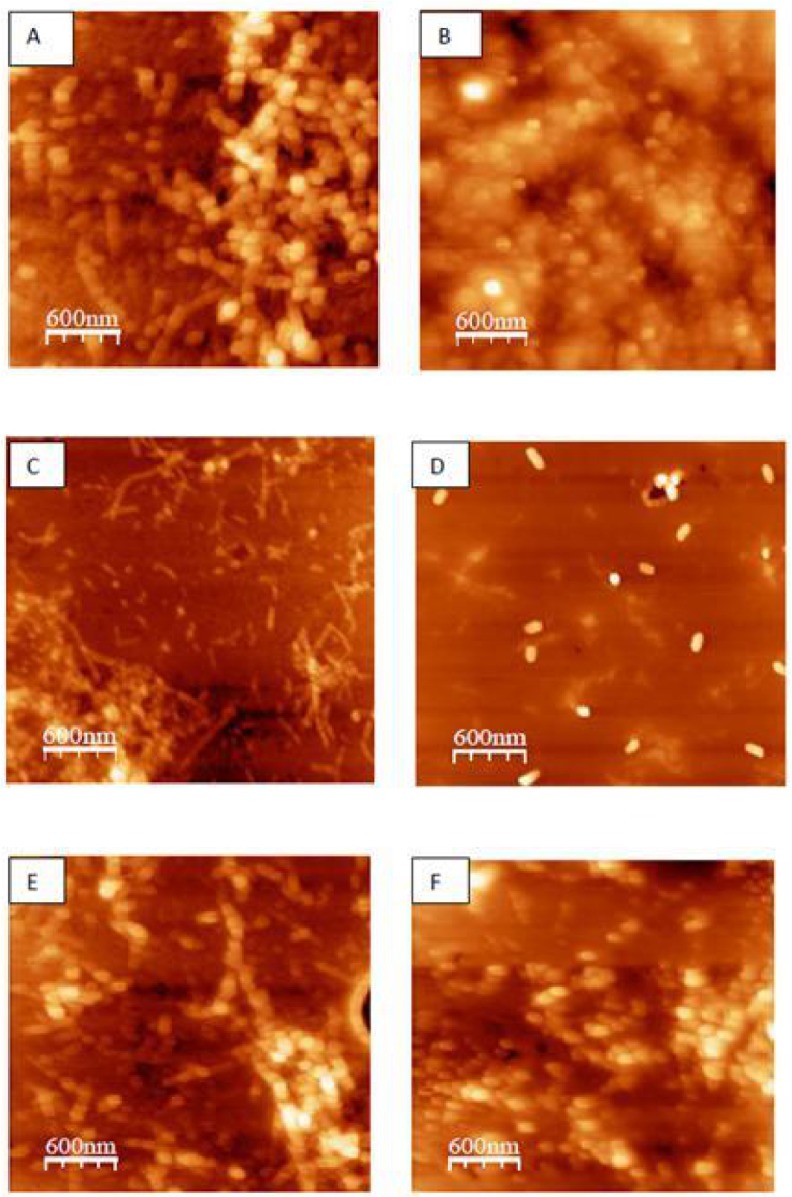
AFM images of different β-Lg samples incubated at 80°C and pH 2 for 24 h. (A) β-Lg alone, (B) β-Lg in the presence of curcumin, (C) β-Lg in the presence of AFM1 (1 ppb), (D) β-Lg in the presence of AFM1 (1 ppb) and curcumin, (E) β-Lg in the presence of Pb^2+^ (0.2 ppm), and (F) β-Lg in the presence of Pb^2+^ (0.2 ppm) and curcumin.

The morphology of the β-Lg fibril samples incubated with curcumin, Pb ions and AFM1 was analyzed by AFM. The AFM image of fibrils (Fig 3) grown at pH 2 and 80 ⁰C does not look like those seen in a number of other studies, because the concentration of used protein is not the same. In higher concentration, more and mature fibrils can be obtained. In many studies the concentration 20 mg/mL or 10 mg/mL of β-lg has been used, but we used 5 mg/mL of protein. Also different researchers reported different structures of β-Lg fibrils [43]. It was found that at low protein concentrations, the incorporation of peptides into fibrils is the rate limiting step for the fibrillar growth [44]. So the fibrils were composed of small peptides, and that other non-aggregated peptides are present in the β-Lg fibril solutions. These structures were observed after incubation for 24 h. The images indicated that fibril did not form in the presence of curcumin (Fig 3B). After co-incubation of β-Lg with AFM1, the amount, diameter, and the length of fibrils were increased after 24 h of incubation (Fig 3C), and was consistent with ThT fluorescence results. In the presence of Pb^2+^, the structure of fibrils is similar Fig 3A (Fig 3E). Fig 3D and 3F show that the effects of AFM1 and Pb^2+^on β-Lg fibril formation were attenuated in the presence of curcumin. Specially, the morphologies of aggregated β-Lg were very different in the presence of curcumin with AFM1 during the incubation, where less fibril was formed under these conditions. These findings indicated that curcumin showed a strong inhibitory effect on the toxin-induced β-Lg fibrillation, and diminished the development of fibrillar amyloids.

### ROS generation and inhibitory effects of curcumin

**Fig 4:**
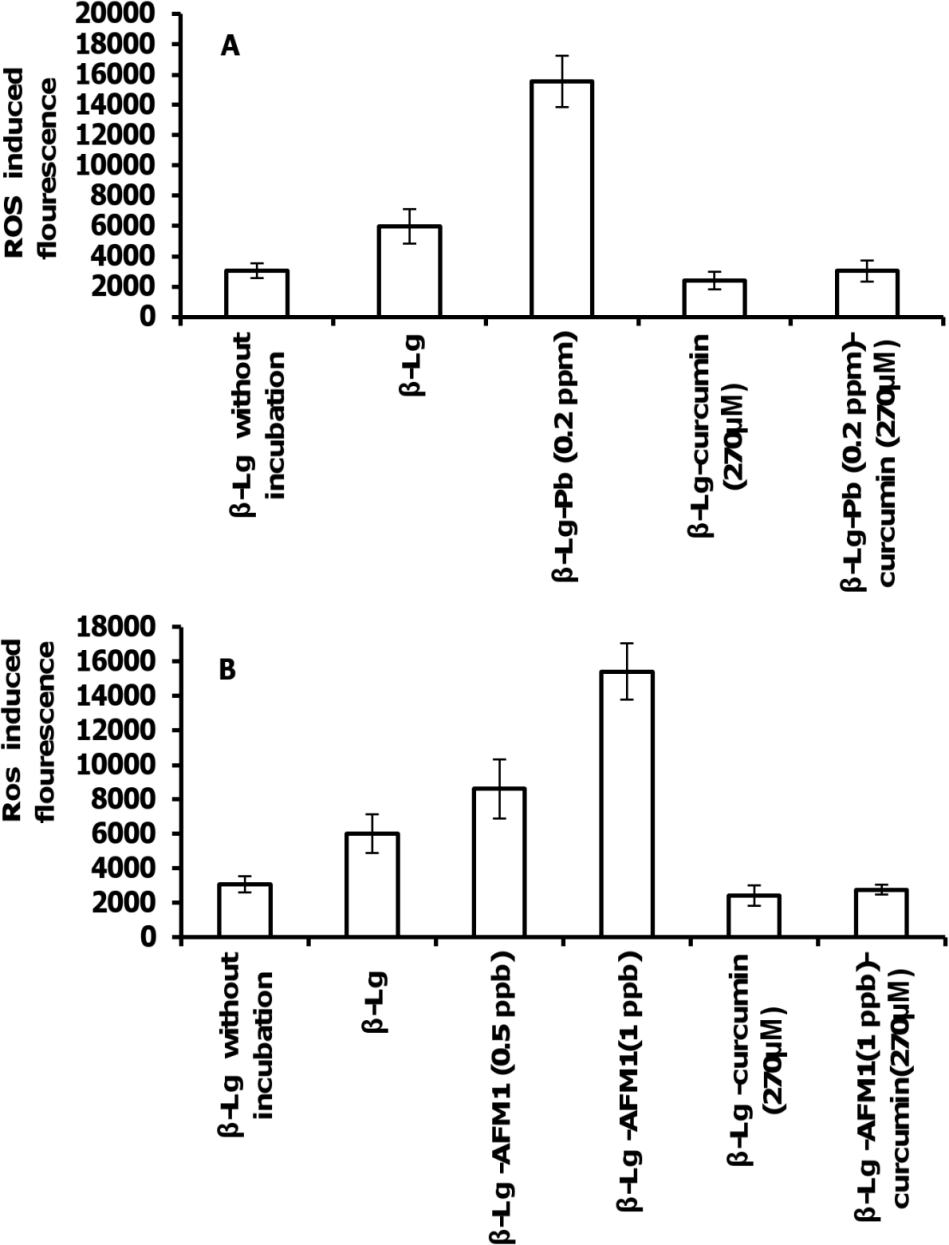
ROS production in β-Lg incubated at 80°C and pH 2. (A) In the absence or presence of curcumin and Pb^2+^, and (B) in the absence or presence of curcumin and AFM1. Data represent the means ± SD of 3 independent measurements.

The impact of curcumin, AFM1, and Pb^2+^ on the level of ROS are shown in Fig 4A and 4B. Fig 4 shows the rate of ROS production of β-Lg incubated at pH 2 and 80°C, in the presence or the absence of curcumin, Pb^2+^ and AFM1.ROS accelerate bril formation perhaps through oxidation reactions. Thus, the free radicals formed during amyloid fibrillization enhanced fibril formation. However, it is unlikely that the amyloid formation is a direct cause or an outcome of ROS formation [45]. Cysteine sulfenic acid (Cys-SOH) is formed by the action of ROS on protein thiols. It is reported that Cys-SOH is formed spontaneously in air-exposed aqueous solution of unfolded (reduced disulfide) proteins in the absence of added oxidizing reagents. Molecular oxygen (O_2_) and trace metals are shown to be important reagents in the oxidative refolding processes. Cys-SOH also plays a role in spontaneous disulfide-based dimerization of peptide molecules containing free cysteine residues [46]. Curcumineffectively decreased ROS levels, consistent with its protective effect in neurodegenerative diseases. We propose that AFM1 and Pb^2+^ promote the formation of proteins fibrils, which are toxic. Fig 4 shows that although both toxins (AFM1 and Pb^+2^) induced ROS generation, curcumin diminished their effects.

### Neurotoxicity in β-Lg fibrils-treated Cells and protective effects of curcumin

**Fig 5:**
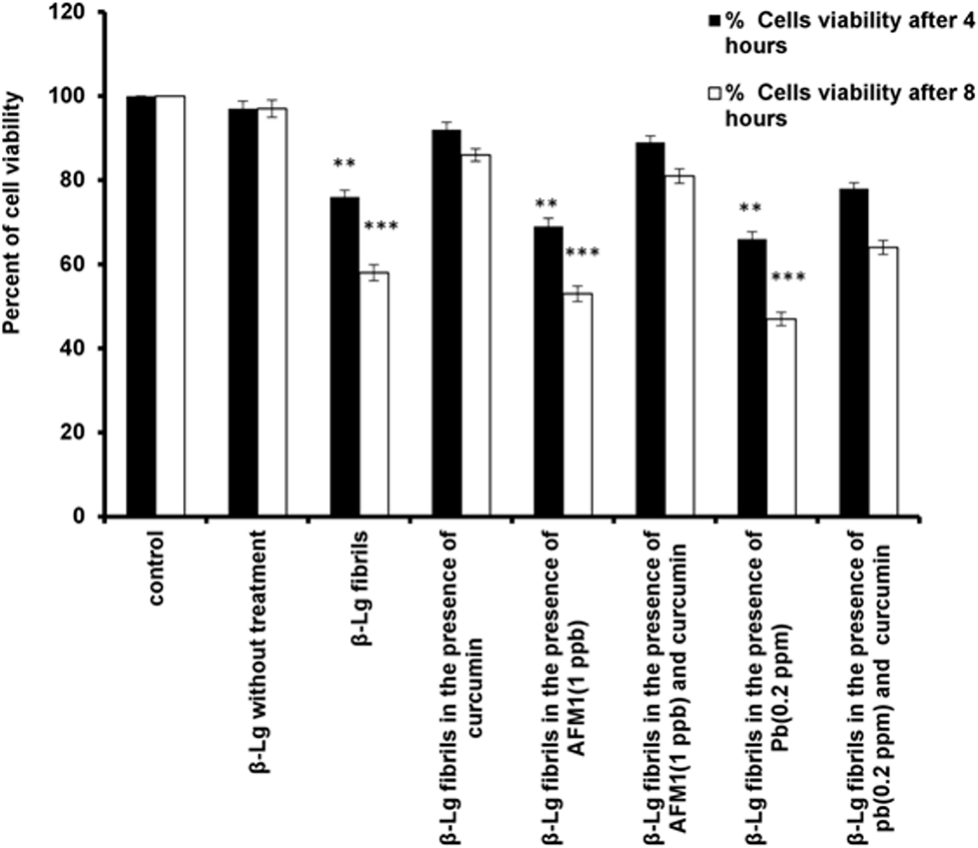
The protective effect of curcuminon β-Lg fibrils-induced cytotoxicity of PC12 cells. PC12 cells were incubated with or without β-Lg fibrils, obtained in the absence and/or presence of curcumin, Pb^2+^, or AFM1. The PC12 cell viability was determined using MTT assay after incubation for 4 or 8 h. Incubation with β-Lg fibrils containing curcumin significantly decreased the amount of cell death compared with β-Lg fibrils in the absence of curcumin. Data represent the means ± S.E.M (n = 3; *significantly different from control).

It is believed that aggregated/ fibrillar amyloid proteins are toxic to neurons and neuron-like cells [47–50]. The PC-12 cells line as a useful model for neurobiological studies have been employed in studying differentiation, survival and apoptosis [51, 52]. Here protein fibrils were employed as a neurotoxicant, and the viability of PC12 cells was measured after incubation with native β-Lgand β-Lgfibrilsobtained in the absence or presence of curcumin, Pb ions and AFM1 using an MTT assay. PC12 cells were incubated with various β-Lg fibril samples for 4 or 8 h. Fig 5 shows that treatment with β-Lg fibrils for 4 or 8 h induced approximately 24% and 42% cell death, respectively, compared with control cells. However, the incubation with β-Lg samples containing curcumin, along with Pb ions or AFM1, increased cell viability by 1.2 and 1.5-fold, respectively, compared with β-Lg fibrils in the absence of curcumin. Incubation of PC12 cells with β-Lg fibrils formed in the presence of Pb^2+^ (0.2 ppm) for 4 or 8 h decreased cell viability by 34% and 54%, respectively, compared with control cells. Incubation of PC12 cells with β-Lg fibrils formed in the presence of Pb^2+^ and curcumin for 4 or 8 h increased cell viability by 1.2 and 1.4 fold, respectively, compared with β-Lg fibrils alone. The incubation of β-Lg fibrils, formed in the presence of AFM1 (1 ppb), for 4 or 8 h decreased cell viability by 31% and 47%, respectively, compared with control cells. However, incubation with β-Lg fibrils formed in the presence of AFM1 and curcumin for 4 or 8 h enhanced cell viability by 1.3 and 1.5 fold, respectively, compared with AFM1 alone.

**Fig 6:**
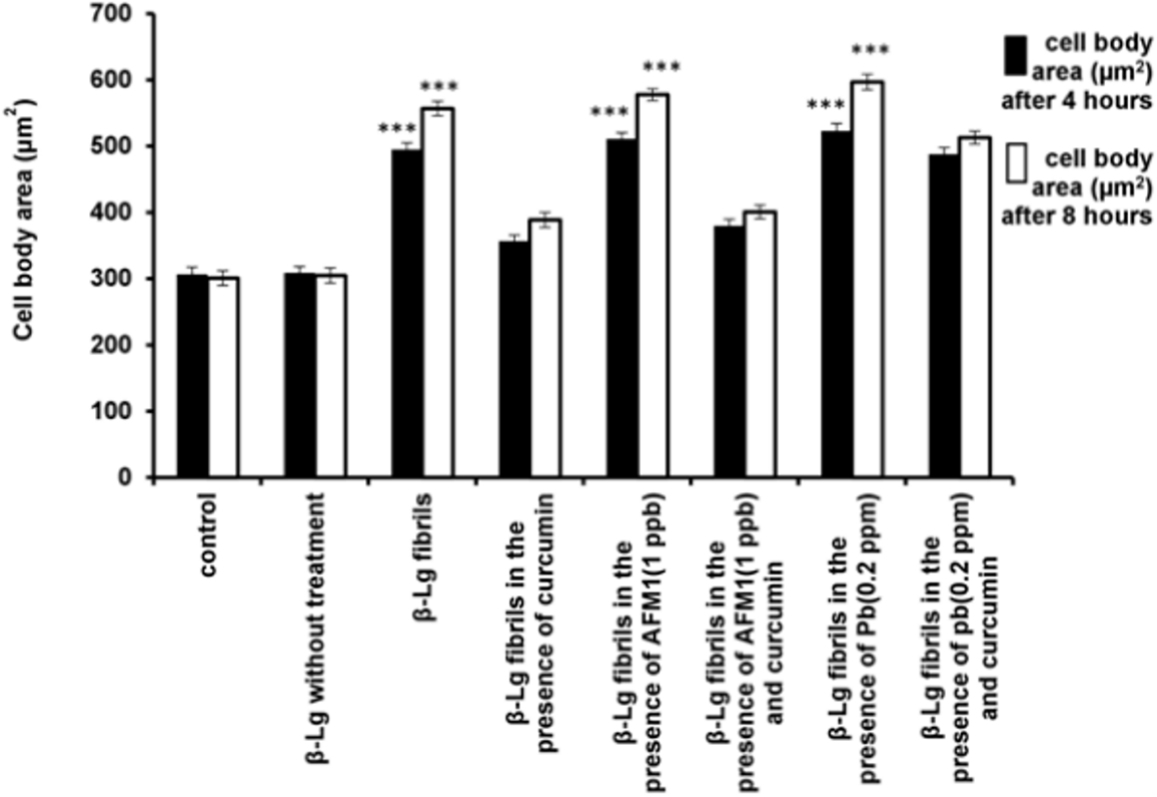
The effects of different forms of β-Lg fibrils on cell body area of neurite outgrowth in differentiated PC12 cells (n = 3; *significantly different from control).

Fig 6 shows that the average cell body area increased in cells incubated with β-Lg fibrils compared with control or native β-Lg. In addition, incubation of cells with β-Lg fibrils, prepared in the presence of Pb ions or AFM1, further increased the average cell body area compared with control cells. However, the incubation of PC12 cells with β-Lg fibril samples formed in the presence of curcumin decreased cell body area by 28% and 30% compared with β-Lg fibrils alone. Furthermore, curcumin compensated for the destructive effects of β-Lg fibrils obtained in the presence of Pb ions or AFM1 on average cell body area of PC12 cells. These averages significantly decreased in the presence of β-Lg fibrils formed in the presence of curcumin compared with incubation with β-Lgfibrils obtained without curcumin.

**Fig 7:**
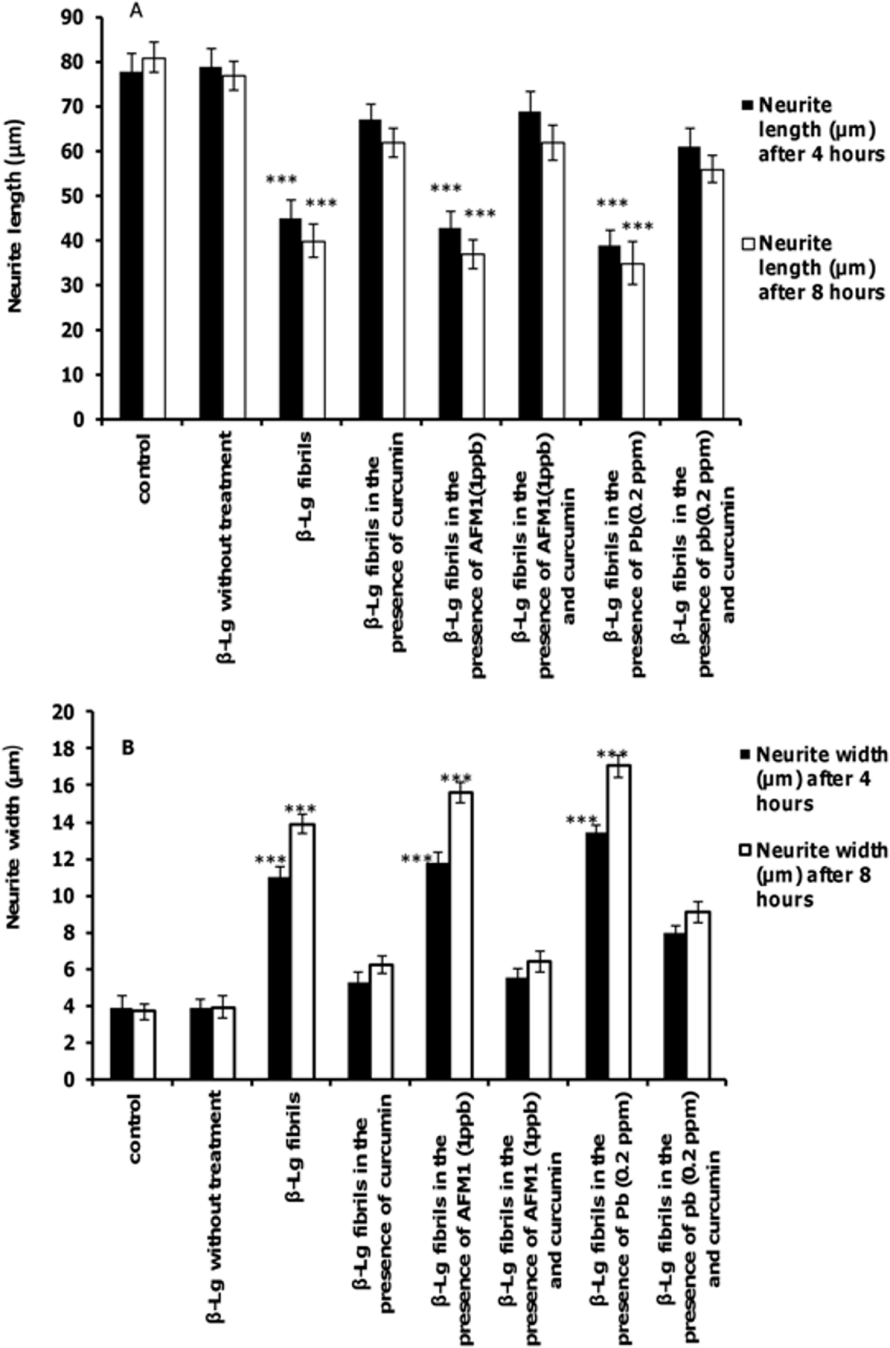
The effects of different form of β-Lg fibrils on neurite outgrowth in differentiated PC12 cells. (A) Average neurite length and (B) average neurite width (n = 3; *significantly different from control).

The effects of test agents on neurite length and neurite width of PC12 cells are shown in Fig 7A and 7B. Neurites in cells exposed to β-Lg fibrils formed in the presence of Pb ions or AFM1 were shorter and wider compared with control cells. However, incubation of cells with protein fibrils formed in the presence of curcumin significantly increased the length of neurons compared with cells exposed to β-Lg fibrils formed in the presence of Pb ions or AFM1 without curcumin. For each criterion, the effect of protein fibrils prepared in the presence of the two toxins was more prominent than the protein fibril formed without these toxins.

**Fig 8:**
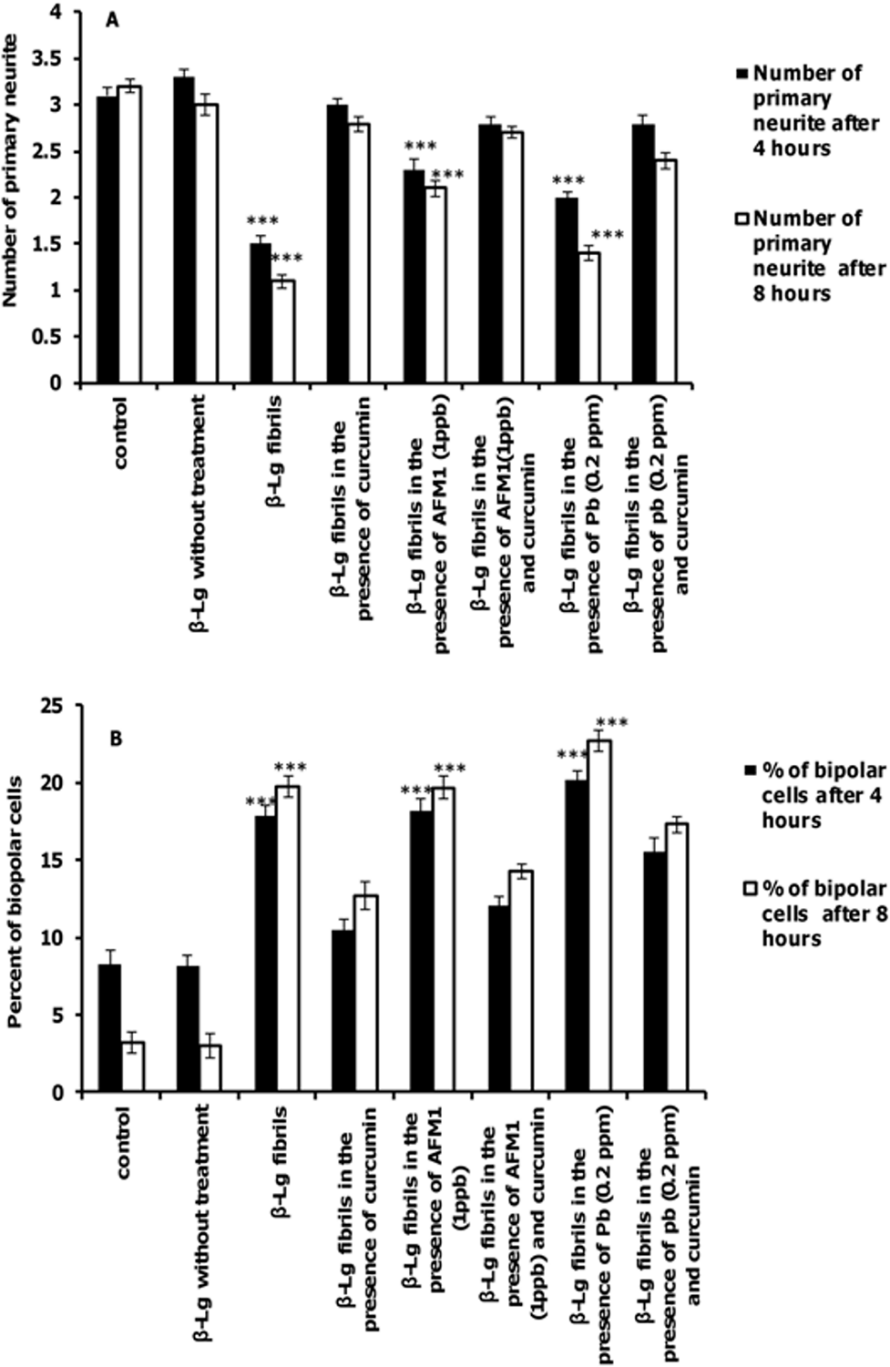
The effects of different forms of β-Lg fibrils on neurite complexity of differentiated PC12 cells. The criteria were quantified after incubation for 4 or 8 h. (A) primary neurites per cell and (B) percent of bipolar cells.(n = 3; *significantly different from control).

As shown in Fig 8A, the number of primary neurites decreased after incubation with β-Lg fibrils formed in the presence or absence of Pb ions and AFM1 compared after incubation with control cells. Incubation of cells with protein samples containing curcumin increased the number of primary neurites relative to β-Lg fibrils formed with and without Pb ions or AFM1. In addition, the percent of bipolar cells incubated with β-Lg fibrils obtained in the presence or absence of Pb ions or AFM1 was significantly increased compared with the control cells. Interestingly, the number of bipolar cells in samples containing fibrils containing curcumin even in the presence of Pb ions or AFM1 is less than cells exposed to fibril samples formed without curcumin (Fig 8B).

**Fig 9:**
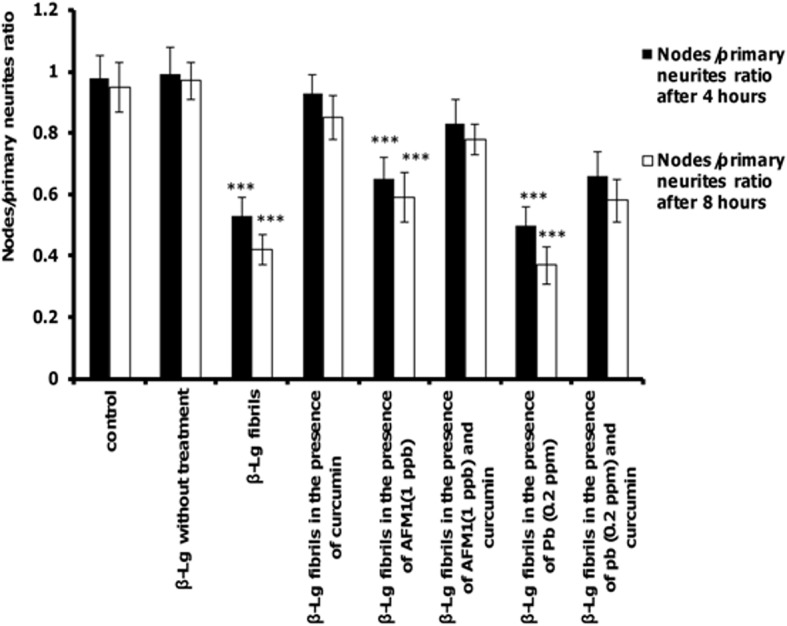
The effects of different forms of β-Lg fibrils on the ratio of nodes to primary neurites of differentiated PC12 cells. The criteria were quantified after incubation for 4 or 8 h. (n = 3; *significantly different from control).

The ratio of total neurite branching nodes to total number of primary neurits was the last parameter of complexity evaluated. Fig 9 shows that the ratio of nodes to primary neurits decreased in cells exposed to β-Lg fibrils formed in the presence and/or absence of Pb ions and AFM1 compared with control cells. However, the presence of curcuminin protein samples significantly increased this ratio compared with non-curcumin-treated protein samples. Reduction of dendrite structure and neuronal complexity are associated with disruption of neuronal function [53, 54]. Thus, all form of β-Lg fibrils can affect the function of PC12 cells by decreasing neuronal dendritic branches, but in the presence of protein samples incubated with curcumin, this effect was negligible.

Collectively, our findings demonstrated that β-Lg fibrils had toxic effects on PC12 cells. Also, the fibrillation process in the presence of food contaminants, such as lead and AFM1, can promote the fibrillation process and cytotoxicity. The extension of incubation times of cells with fibrils would probably show more cytoprotective effects. However, curcumin inhibited protein fibril formation and protected PC12 cells against fibril-induced cytotoxicity.

## Conclusions

Regarding fibril formation, some food proteins have received increased attention because of their properties in food industry. Assembly of proteins in to fibrils is an important subject of study in various aspects of human health. Despite of great attention to fibrillation of whey proteins and using them in industry, β-Lg fibrils are shown to be toxic to neuronal cells in culture and cause free radicals formation. The existence of some contaminants such as lead and aflatoxin mediates the formation of free radicals causing various modifications in protein structure, and may induce neurodegenerative diseases. Thus, it is important to use food protein fibrils with more caution. Our results also suggest that the intake of curcumin may potentially provide protection against the cytotoxicity of fibrils, and it may act as a neuroprotective agent in neurodegenerative disorders.
